# Aerosol optical depth (AOD): spatial and temporal variations and association with meteorological covariates in Taklimakan desert, China

**DOI:** 10.7717/peerj.10542

**Published:** 2021-01-05

**Authors:** Jinglong Li, Xiangyu Ge, Qing He, Alim Abbas

**Affiliations:** 1 College of Resources and Environment Sciences, Xinjiang University, Urumqi, China; 2 Institute of Desert Meteorology, China Meteorological Administration, Urumqi, China; 3 Key Laboratory of Oasis Ecology, Xinjiang University, Urumqi, China; 4 Key Laboratory of Smart City and Environment Modelling of Higher Education Institute, College of Resources and Environment Sciences, Xinjiang University, Urumqi, Xinjiang, China

**Keywords:** Aerosol optical depth (AOD), Dust aerosol, Spatiotemporal distribution, Impact factors

## Abstract

Aerosol optical depth (AOD) is a key parameter that reflects aerosol characteristics. However, research on the AOD of dust aerosols and various environmental variables is scarce. Therefore, we conducted in-depth studies on the distributions and variations of AOD in the Taklimakan Desert and its margins, China. We examined the correlation characteristics between AOD and meteorological factors combined with satellite remote sensing detection methods using MCD19A2-MODIS AOD products (from 2000, 2005, 2010, and 2015), MOD13Q1-MODIS normalized difference vegetation index products, and meteorological data. We analyzed the temporal and spatial distributions of AOD, periodic change trends, and important impacts of meteorological factors on AOD in the Taklimakan Desert and its margins. To explore the relationships between desert aerosols and meteorological factors, a random forest model was used along with environmental variables to predict AOD and rank factor contributions. Results indicated that the monthly average AOD exhibited a clear unimodal curve that reached its maximum in April. The AOD values followed the order spring (0.28) > summer (0.27) > autumn (0.18) > winter (0.17). This seasonality is clear and can be related to the frequent sandstorms occurring in spring and early summer. Interannual AOD showed a gradually increasing trend to 2010 then large changes to 2015. AOD tends to increase from south to north. Based on the general trend, the maximum value of AOD is more dispersed and its low-value area is always stable. The climatic index that has the most significant effect on AOD is relative humidity.

## Introduction

Atmospheric aerosol is a general term used in a multi-phase system comprising solid or liquid particles suspended in gas. Aerosols are vital trace materials in the atmosphere (Baltensperger & Prévôt, 2008). Particle sizes range between 10^−3^ and 10^2^ μm, and its total mass accounts for only one billionth of the entire atmospheric mass. As a basic optical parameter, aerosol optical depth (AOD) is a measure of the extinction effect of atmospheric aerosols and is widely used as a key parameter for assessing the degree of air pollution. Moreover, aerosols have been analyzed in research on climate change and atmospheric radiation balance. Therefore, aerosols have a profound impact on local, regional, and even global climate as well as atmospheric radiation transmission and water cycles (Charlson et al., 1991; Rosenfeld et al., 2007). Dust aerosol is an important component of atmospheric aerosol and accounts for more than 50% of the total aerosol content (Andreae, 1995). In the troposphere, the proportion of atmospheric aerosols reaches one-third (Harrison et al., 2001). A change in tropospheric aerosols result in a strong heating or cooling effect and can change the thermal state of the atmosphere, which affects the dynamic structure of the atmosphere (Huang et al., 2007), thereby leading to climate change. When sandstorms occur, high winds drag large amounts of dust into the air and cause serious local pollution. Some finer dust particles are lifted up to high altitudes and travel long distances with the upper airflow, which affects the air quality over a large area. Long-distance transportation of particles by sand and dust storms is considered an important part of the global biogeochemical cycle (Zhuang et al., 1992). Meteorological elements can affect aerosols. Satellite and aerial imaging observations confirm that cloud droplets containing dust aerosols rarely cause precipitation (Rosenfeld, Rudich & Lahav, 2001). Therefore, clarifying the relationship between dust aerosols and meteorological factors is helpful for predicting the impact of dust aerosols on the global climate change and global biogeochemical cycle.

Currently, remote sensing (RS) technology is an important means to detect and monitor aerosol based on the AOD (Badarinath et al., 2011; Ge et al., 2011; Ginoux et al., 2001; Huang et al., 2007, 2009; Masoumi et al., 2013; Perry, Cliff & Jimenez-Cruz, 2004; Sever et al., 2017; Tegen & Fung, 1994; Wang et al., 2010). As a column-integrated quantity, AOD reflects aerosol column loading and the impact of aerosols on Earth’s radiation budgets (Park, Ahn & Chang, 2007). Owing to its unique advantages, RS provides a feasible AOD acquisition method with high temporal resolution on a wide range of spatial scales. RS can overcome the problems related to the lack of ground observation data and uneven spatial distribution. Further, RS provides a reference for a comprehensive understanding of aerosol concentration and distribution as well as theoretical support for regional atmospheric environment management. AOD information retrieved by MODIS provides good global distribution and daily or nearly continuous time coverage. MODIS products were used to analyze the monthly variation characteristics of East Asian aerosol optical properties from 2000 to 2005 (Kim et al., 2007). The Terra-MODIS aerosol product (MOD04) was used to retrieve the aerosol characteristics in Xinjiang, and the retrieval algorithm was found to have limitations (Tian et al., 2018). However, these MODIS products do not have a fine resolution, only 1°, 0.5° and 3 km pixel resolution. There are also concerns regarding the accuracy of the inversion products. The recently released MCD19A2 product integrates Terra and Aqua and uses the multi-angle implementation of atmospheric correction (MAIAC) algorithm. They yield AOD products exhibiting high resolution (1 km pixel resolution), wide inversion range, and high inversion accuracy. However, it has not been evaluated with observational data at the same scale, particularly in the regions with scarce data. Therefore, it is essential to verify the product and conduct a spatiotemporal analysis based on the product.

The Taklimakan Desert is the largest desert in China which contains extremely complex dune types located in the center of the Tarim Basin. It is the second largest mobile desert in the world and one of the main sources of dust aerosol in China. Fine particulate matter and ozone are the primary causes of pollution in the basin (Zhao et al., 2018). The Cloud-Aerosol Lidar with Orthogonal Polarization inversion reveals that the height of the dust aerosol layer in the Taklimakan Desert reaches 4–5 km, and the vertical distribution of color ratio and particle depolarization shows that dust plays a dominant role (Zong, Xia & Che, 2015). The AOD over the Taklimakan Desert is higher in spring and summer and lower in autumn and winter. The frequency distribution of AOD and the probability of the Angstrom exponent are unimodal (Che et al., 2013). In 2016, the air pollutants in southern Xinjiang showed obvious seasonal and spatial distribution characteristics; moreover, During sandstorm process, the concentration of PM10 and PM2.5 will increased abruptly (Yu et al., 2019). PM10 is the main pollutant affecting air quality in western China (Zhang et al., 2017). A study on atmospheric dust fall in Urumqi, China, showed that the total suspended particulate matter (TSP) is the main pollutant, and the northwest wind in spring and summer can bring dust particles from the desert to this region (Zhang et al., 2014). The increase in the aerosol load in Central Asia can be attributed to aerosols transported from the arid area, which is affected by precipitation and dust; the aerosol contribution to this region is significant in spring and summer (Rupakheti et al., 2019). Many types of aerosols are present in Dushanbe, Tajikistan, and aerosols in the atmosphere can significantly impact the climate of Central Asia, Tianshan Mountains, Tibet Plateau, and other regions (Rupakheti et al., 2020). The AOD in the Republic of Kazakhstan shows a clearly increasing trend, which is primarily affected by the aggravation of desertification (Kumar et al., 2018). These results clarify that aerosols in Central Asia show a rising trend. Whether the diffusion and migration of dust aerosols from the center of Tarim Basin to central Asia will affect the aerosol load and climate of Central Asia has practical significance. However, there is limited research on the close relationship between dust aerosol and meteorological factors. The Taklimakan Desert has the most complex dune types, the most mobile sand, the largest proportion of mobile desert area, the thickest mobile sand layer, the finest sand grains and other characteristics, and its natural characteristics are typical in deserts all over the world. Therefore, the study of the temporal and spatial distribution of dust aerosols and their relationship with meteorological elements has an important impact on climate change and ecological environment in the areas surrounding northwest China.

The objectives of this research are to (1) study the change trend and the trend of aerosols in four periods (2000, 2005, 2010, and 2015) and their relationship with MODIS normalized difference vegetation index (NDVI) and meteorological factors, (2) analyze the effect of meteorological factors on AOD, and (3) explore the relevant characteristics of aerosols in the Taklimakan Desert and its margins.

## Materials and Methods

### Overview of the study area

The Taklimakan Desert is located in the Tarim Basin of Xinjiang, China. It is the largest mobile desert in China and the second largest in the world. The Taklimakan Desert has an extremely dry climate, sparse vegetation, and scarce rainfall. It has rich sand sources and maximal bare ground and only scattered tamarix shrubs and reeds are distributed between the ridges. It mainly comprises flowing sand, dry soil, loose structures, and exposed surfaces with flowing shapes that are prone to wind erosion and dusty weather. Easterly winds prevail throughout the year with an annual average of more than 157 days of dust weather and approximately 16 days of sandstorms. The dust and aerosol concentrations in the atmosphere are considerably higher in this region than in cities.

### Satellite RS data

#### MCD19A2-MODIS AOD data

The MODIS sensor has 36 discrete spectral bands with a spectral range of 0.41–14.5 µm and covers a geographic range of 1,200 km × 1,200 km with a spatial resolution of 1 km. These features are realized using the aerosol algorithm in MAIAC, which can accurately and effectively retrieve AOD data from lands and oceans. The latest MODIS aerosol products provide accurate algorithms for inverting aerosol characteristics from lands and oceans, particularly the extended dark blue algorithm that is suitable for bright undersurfaces, such as deserts, plateaus, and arid regions (Sayer et al., 2014; Levy et al., 2013). The inversion accuracy is high, and the accuracy of dark surfaces is similar to that of the dark target method. The accuracy of bright surfaces is better than that of the dark blue algorithm. This study uses MCD19A2 AOD daily product data. LAADS DAAC (https://ladsweb.modaps.eosdis.nasa.gov/search/) downloads of MODIS aerosol data products from 2000, 2005, 2010, and 2015 are also used. The downloaded data are projection converted and mosiaced using the MRT tool. The AOD data of each month are synthesized using daily averages to ensure ground surface reflectivity as close to the real situation as possible. Considering pixels as the processing unit, the mean value of effective pixels (ignoring the filling and error values) is calculated. The corresponding AOD year, month, and season values were obtained by vector cropping the Taklimakan Desert and its margins and finally plotting using ArcMAP software.

#### MOD13Q1-MODIS NDVI data

MODIS NDVI data complements NOAA’s AVHRR NDVI product and provides continuity for the application of time series in this rich historical archive. MODIS NDVI data products are calculated based on the atmospherically corrected two-way surface reflectance values that have obscured the shadows of water, clouds, and heavy aerosols. The MOD13Q1 data product (LAADS DAAC) downloaded for use in this study has a spatial resolution of 250 m. Data are provided every 16 days. The monthly data are composed of two 16-day data. The data are transformed, cropped, and synthesized with AOD data. They are merged with the meteorological data in time series.

### Meteorological data

Meteorological data are provided by the Tazhong station. The data include average wind speed, air temperature, relative humidity, and evaporation. According to the classification standard of the general meteorological classification method, the months of March–May are classified as spring, June–August summer, September–November autumn, and December–February winter. These divisions correspond to the time scale of downloaded MODIS AOD data. Weather data for each of the four intervals of 2000, 2005, 2010, and 2015 were selected (for a total of 48 months), and averages from March to May, June to August, September to November, and December to February calculated. The average values calculated were seasonal and annual average temperature, average relative humidity, average wind speed (the data is recorded every 2 s), maximum wind speed, and evaporation.

### Ground-based AOD data

**Figure 1:**
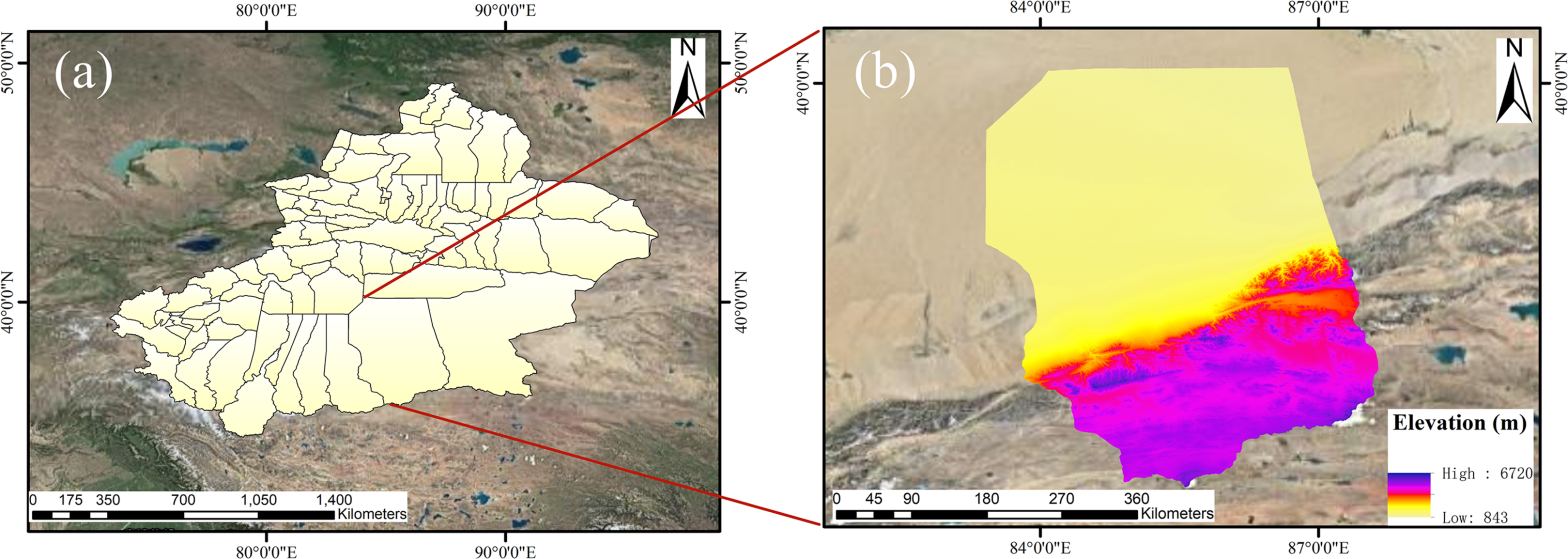
Location of study area. Study area reference Qiemo County. (A) The complete area of Xinjiang. (B) Map of the study area.

A CE-318 sun sky lunar multispectral photometer is located in the Tazhong Desert Atmospheric Environment Observation Experimental Station of the China Meteorological Administration (38°58′N, 83°39′E, altitude 1,090 m) as shown in Fig. 1. This meteorological station is nearly 200 km deep in the Taklimakan Desert and monitors desert weather and climate.

An 8-band CE-318 sun sky lunar multispectral photometer (Cimel Electronique, Paris, France) was used for AOD observations in this study. This instrument can measure field solar and sky radiations with high accuracy, including 440-, 670-, 870-, 1,020-nm aerosol bands, 936-nm water vapor band, and three 870-nm polarimetric bands. The bandwidth of each channel is 10 nm. The observation time was from March to December 2015 and observations were not conducted in November. The ground-based AOD data correspond to the average data 30 min before and after the satellite transit time. The daily sun photometer data were integrated to obtain monthly ground-based AOD data. These data were then used to obtain the AOD value in the 550-nm band using the Angstrom exponent calculation, compared with the AOD values of the MODIS products, and used to verify AOD data of the MODIS products. The AOD value is calculated as follows:

$$\tau  = \beta \varepsilon  - \alpha $$

where τ(ε) is the AOD, α is the Angstrom exponent that represents the proportion of large or small particles in the aerosol component, and β is the Angstrom turbidity coefficient that measures the aerosol concentration in the atmosphere.

The Angstrom exponent calculation formula is as follows:

$$\alpha  = {{\ln [\tau (\lambda 1)/\tau (\lambda 2)]} \over {\ln (\lambda 1/\lambda 2)}}$$

where τ(λ) is the AOD and λ_1_ and λ_2_ are the wavelengths (nm).

### Random forest model

The random forest (RF) algorithm is a machine learning algorithm for classification and regression. It integrates multiple decision trees using the concept of holistic learning. The RF algorithm can process high-dimensional data and can be used in collections. It uses a large number of trees (Sun et al., 2018). Partition variables between tree correlations in the minimized set are randomly selected using RF for regression, and the output is the average of the outputs from all decision trees (Chen et al., 2019; Wang et al., 2018). The RF method utilizes many variables and few samples. It can rank the control factors of variables and provide relative importance of each variable (Hong et al., 2019). Moreover, for unbalanced samples, RF can balance errors and has its own advantages of avoiding overfitting and reducing generalization errors. In this study, the AOD was the dependent variable and six environmental covariates, such as average temperature, average relative humidity, average wind speed, maximum wind speed, evaporation, and NDVI, were the independent variables. We used environmental variables to estimate the AOD value and obtained the importance of environmental covariate under the framework of the RF model.

## Results and Analysis

### MODIS product verification

**Figure 2:**
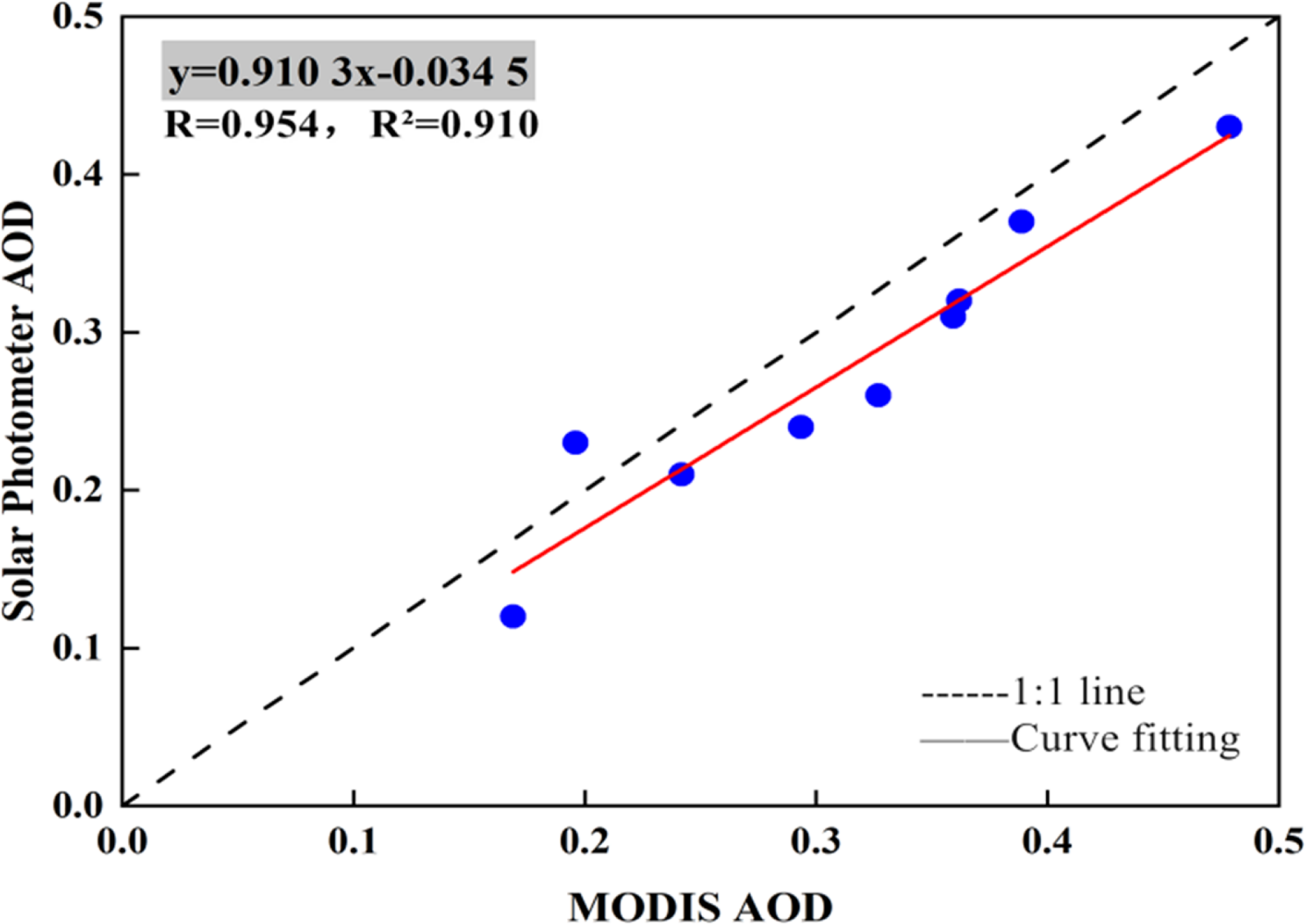
Comparison of MODIS AOD product data with ground-based observations by solar photometers.

The results of the MODIS aerosol products (MCD19A2) were verified by comparing them with measured data obtained using a CE-318 solar photometer (Fig. 2), obtaining *R* = 0.954 and *R*^2^ = 0.910. The fitting curve is nearly 1:1. The line height is consistent with high precision. Based on these results, the quality of AOD data calculated using the MODIS AOD product is considered reliable.

### Spatiotemporal distributions of AOD

**Figure 3:**
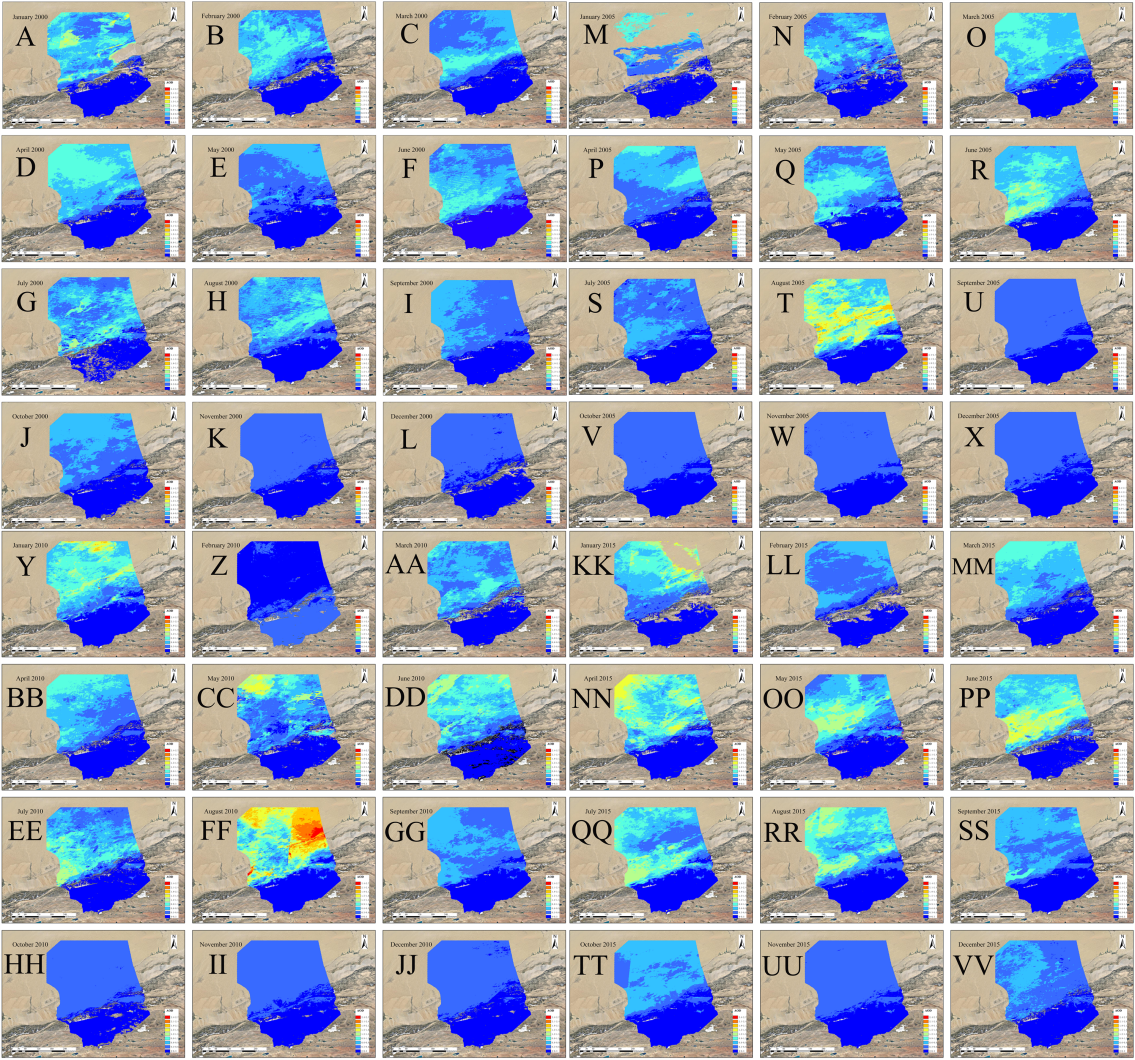
AOD spatial distributions of each month in 2000/2005/2010/2015. (A–L) January to December of 2000, (M–X) January to December of 2005, (Y–J) January to December of 2010, and (KK–VV) January to December of 2015.

The average AOD spatial distribution in the Taklimakan Desert of each month in 2000, 2005, 2010, and 2015 is shown in Fig. 3. The low-value area of AOD in the southern part of the study area and its margins is relatively stable over time. High-value areas occupy the center of the Desert and its northern margins. The spatial distribution of AOD is mainly affected by topographical factors and shows an obvious step-up from south to north.

The white areas in the picture are null values. The inversion algorithm of MODIS AOD uses the dark pixel algorithm. Because snow covers the dust particles, the ground surface brings a bright background, this situation causes a change in ground surface reflectivity. Hence, the AOD value is missing in winter.

The AOD value of each month in these four periods ranged from 0.43 to 2.41 and show that the change in AOD is a flow-cycle process. In this study, the AOD for the 12 months of 2000, 2005, 2010, and 2015 were processed in a unified manner and changes were analyzed to support attribution studies assessing the role of climate variability and change on the Taklimakan Desert.

**Figure 4:**
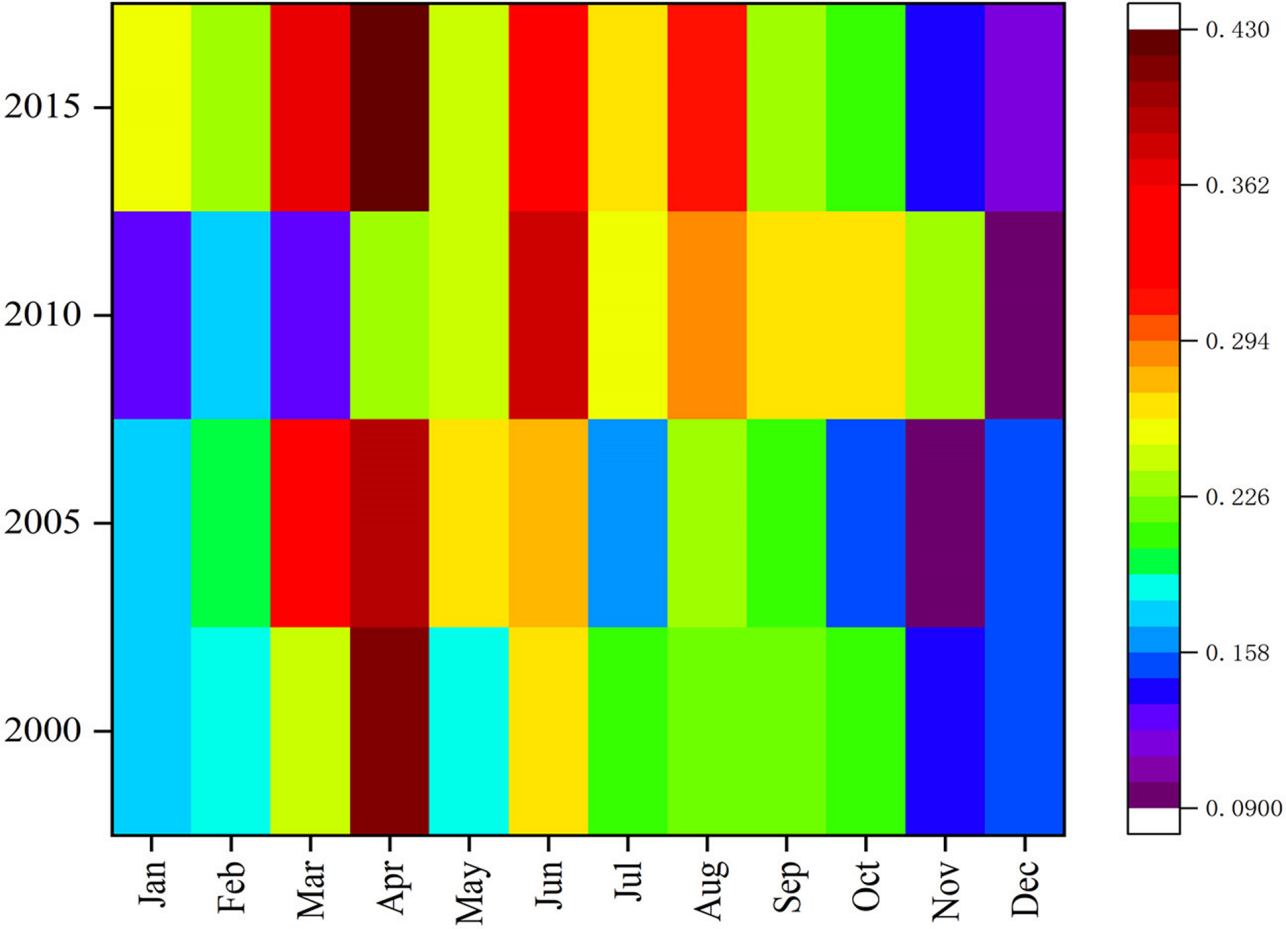
AOD data heat maps for each month of 2000/2005/2010/2015. The labels show years (2000, 2005, 2010, 1015) and months (January, February, March, April, May, June, July, August, September, October, November, and December).

**Figure 5:**
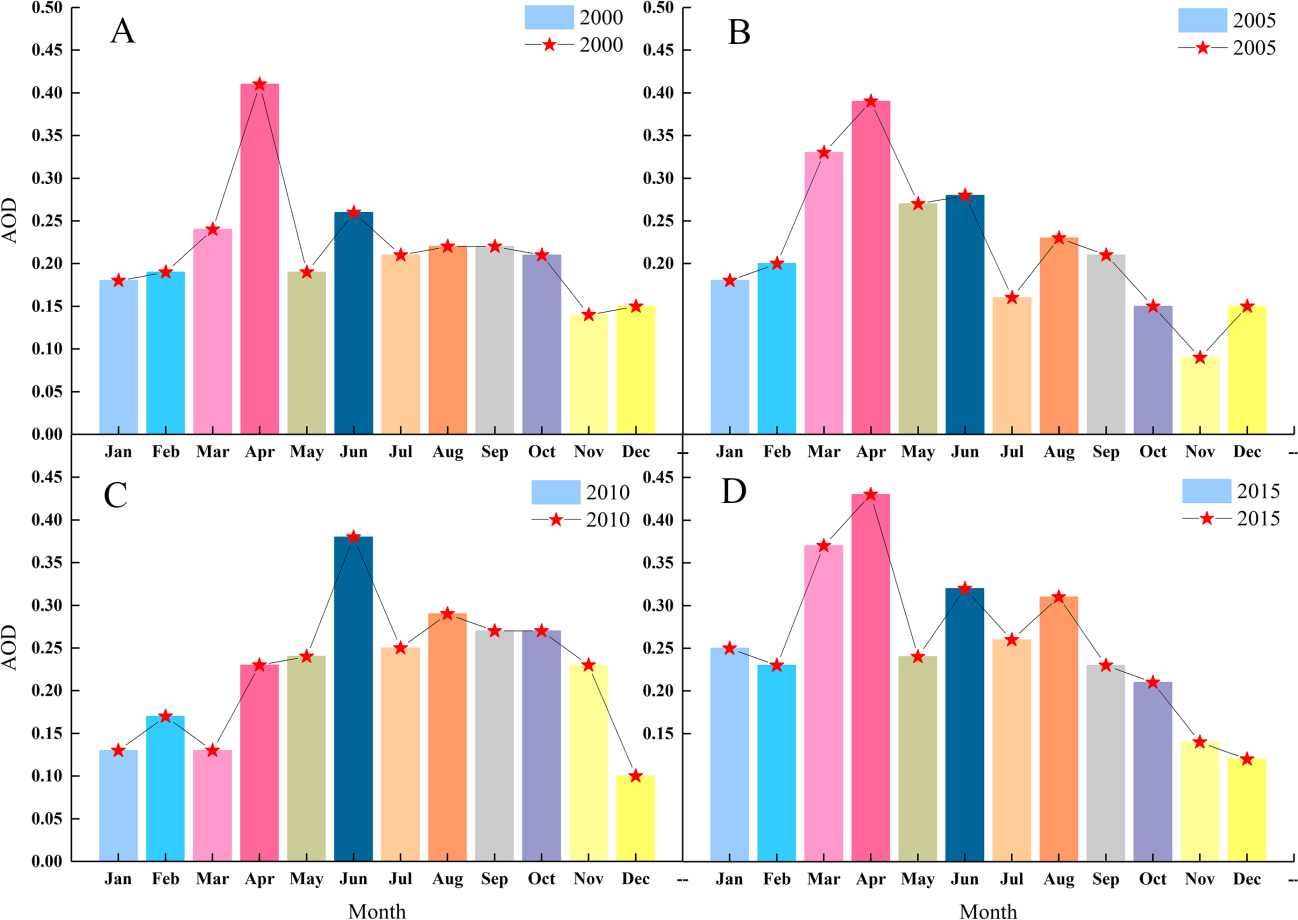
Average AOD in each month of 2000 (A), 2005 (B), 2010 (C) and 2015 (D). The labels show AOD values and months (January, February, March, April, May, June, July, August, September, October, November, and December).

After the MODIS AOD data was processed, the average AOD for each month in 2000, 2005, 2010, and 2015 was calculated, analyzed and compared. The spatial distribution of AOD data was similar in all four periods. In January, February, September, November, and December, Fig. 4 shows the hot spot colors did not change significantly for the medium- and low-values. However in the other moths AOD values varied widely. Based on changes in color distribution, the AOD values of the four periods show a clear seasonal cycle. The high and low AOD values were mainly observed in April and winter, respectively. As shown in Fig. 5, the AOD values for the 12 months of 2000, 2005, 2010, and 2015 followed a similar seasonal pattern of decreasing-increasing-decreasing trend, expressed as an inverted “W.” Except for June 2010 (0.38), the highest values of AOD occurred in April 2000, 2005, and 2015 (0.41, 0.39, and 0.43, respectively) and low values all occurred in November or December. The AOD value reached its maximum in April–June. After October, cold air entered from the north and moved east to south, causing the weather to become colder. Although the main effect of cold air did not fully enter the desert and its margins, the desert was still covered by snow dusty weather decreased because of the southward path of cold air; this further reduced the AOD values. The four sets of AOD RS data show that monthly AOD values are mainly affected by the weather.

### Seasonal time series of AOD

**Figure 6:**
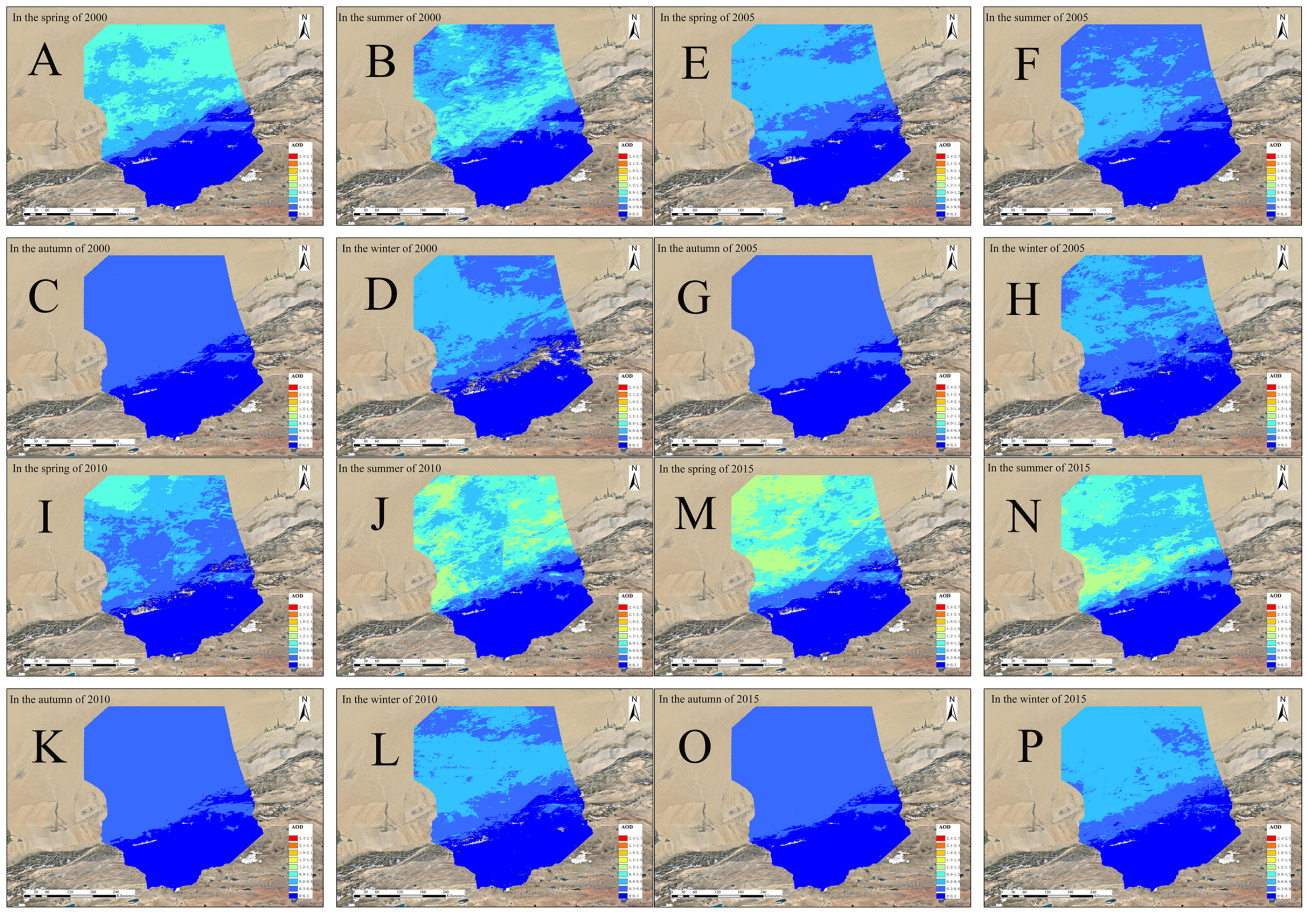
Spatial distributions of AOD in the four seasons of 2000/2005/2010/2015. (A–D) Spring, summer, autumn and winter of 2000, (E–H) spring, summer, autumn and winter of 2005, (I–L) spring, summer, autumn and winter of 2010, and (M–P) spring, summer, autumn and winter of 2015.

**Figure 7:**
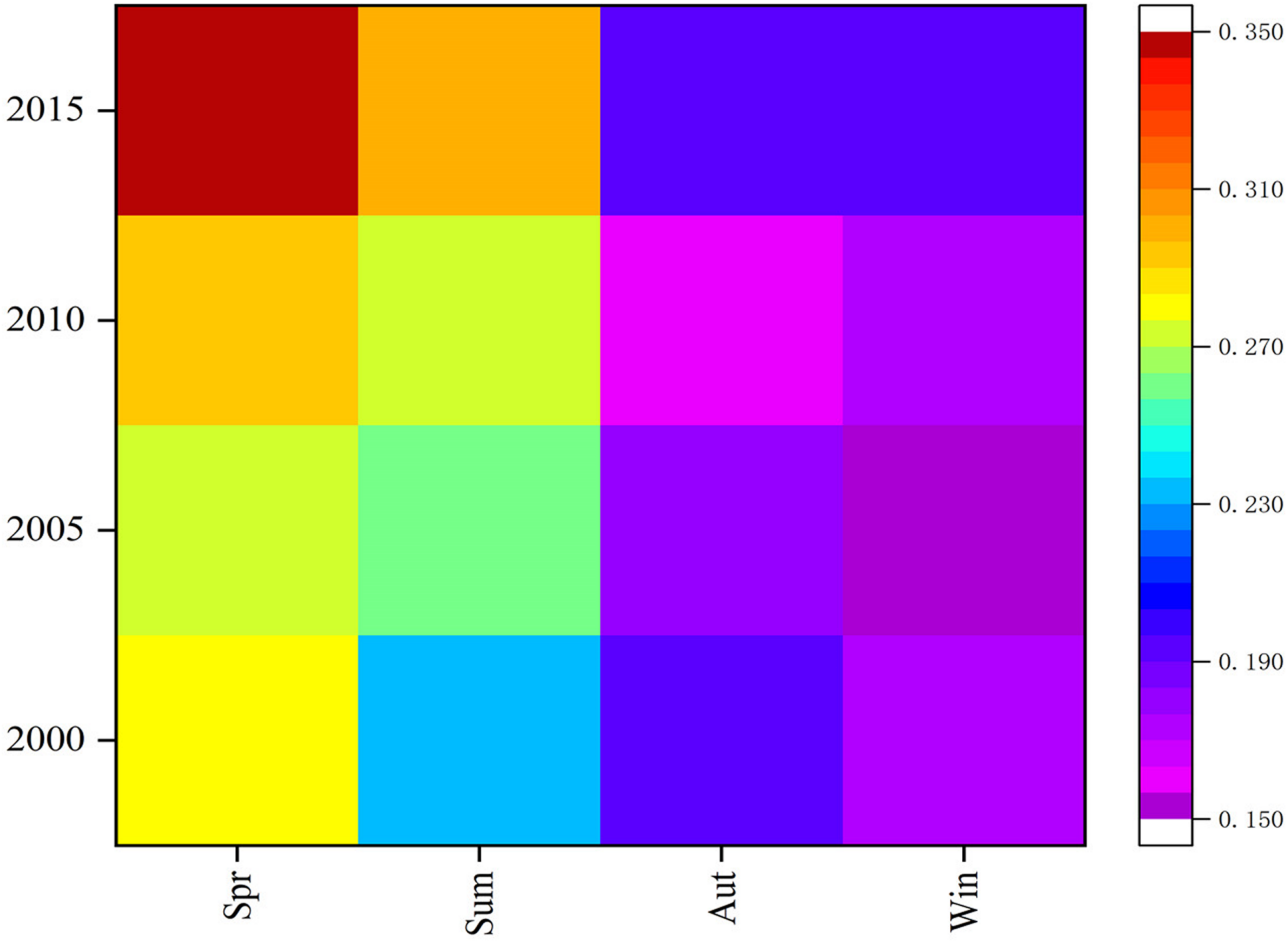
AOD data heat maps for the four seasons of 2000/2005/2010/2015. The labels show years (2000, 2005, 2010, 1015) and the seasons (spring, summer, autumn and winter).

**Figure 8:**
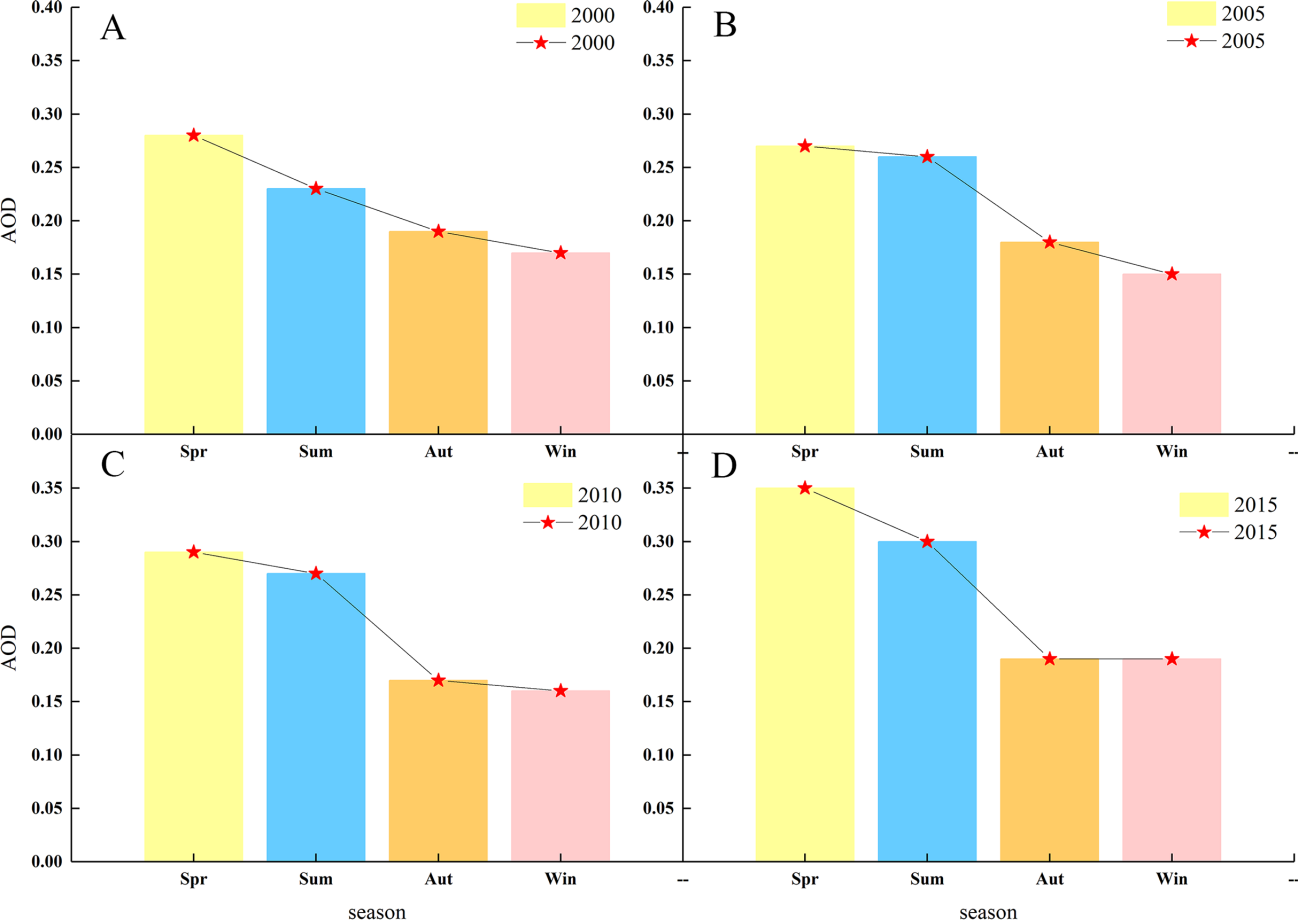
Average AOD for each season 2000 (A), 2005 (B), 2010 (C) and 2015 (D). The labels show AOD values and seasons (spring, summer, autumn and winter).

Figure 6 shows the spatial AOD distributions for the four seasons of 2000, 2005, 2010, and 2015, with high values in the north and low in the south. The low-value area shows little spatial variation, while the high-value areas show much more variation but are relatively evenly distributed in the middle and upper parts of the desert and its margins. From Fig. 7, the annual variation of AOD in 2000, 2005, 2010, and 2015 are almost the same. AOD values in spring and summer increased between 2000 and 2015, with the largest increase in spring. In autumn and winter, AOD value ranges from 0.150 to 0.230, without showing significant change. The seasonal cycle is relatively stable. High AOD occurs in spring and is often associated with sandstorm events occurring in this season. Wind speeds accelerate the movement of mobile dunes; upward air accumulation and associated dust on natural surfaces lead to the highest AOD concentrations in the Taklimakan Desert in spring. Figure 8 shows the highest and lowest AOD values occurred in spring and winter, respectively. There is a gradual decrease from spring to winter in 2000, and the decrease from summer to autumn becomes more abrupt over time. Only spring and early summer are accompanied by large numbers of dusty days, while dusty weather lessens in late summer and autumn with AOD values decreasing. The AOD values for all four seasons from 2000, 2005, 2010 and 2015 were below 0.4 with those in spring being highest (0.28, 0.27, 0.29, and 0.35, respectively). Low AOD values in winter is attributed to reduced winter wind and dust activity, fewer sandstorms, and snowfall.

### Annual averages with 5-year intervals and seasonal variations of AOD in the temporal and spatial changes in the Taklimakan Desert and its margins

**Figure 9:**
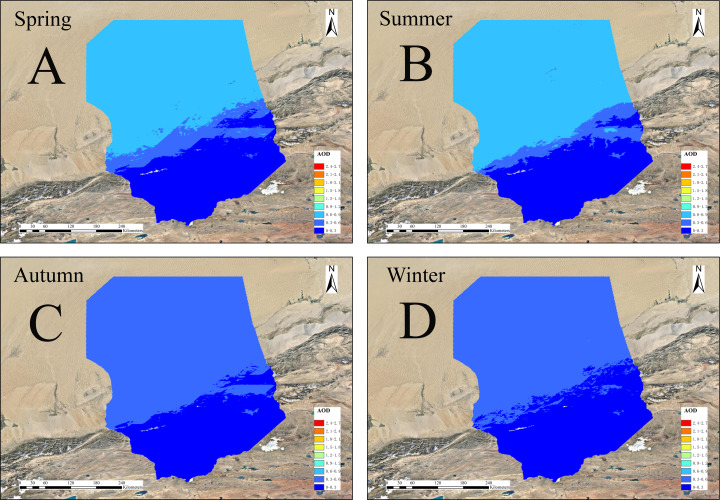
Seasonal mean AOD distribution. (A–D) Spring, summer, autumn, and winter.

Figure 9 shows spatial distributions of average AOD in the four seasons of the Taklimakan desert from 2000 to 2015. They are all at the bottom of the desert and its margins. The trend is particularly obvious in autumn; the range of high values in summer and winter is small and scattered and gradually collapses in the outer areas.

**Figure 10:**
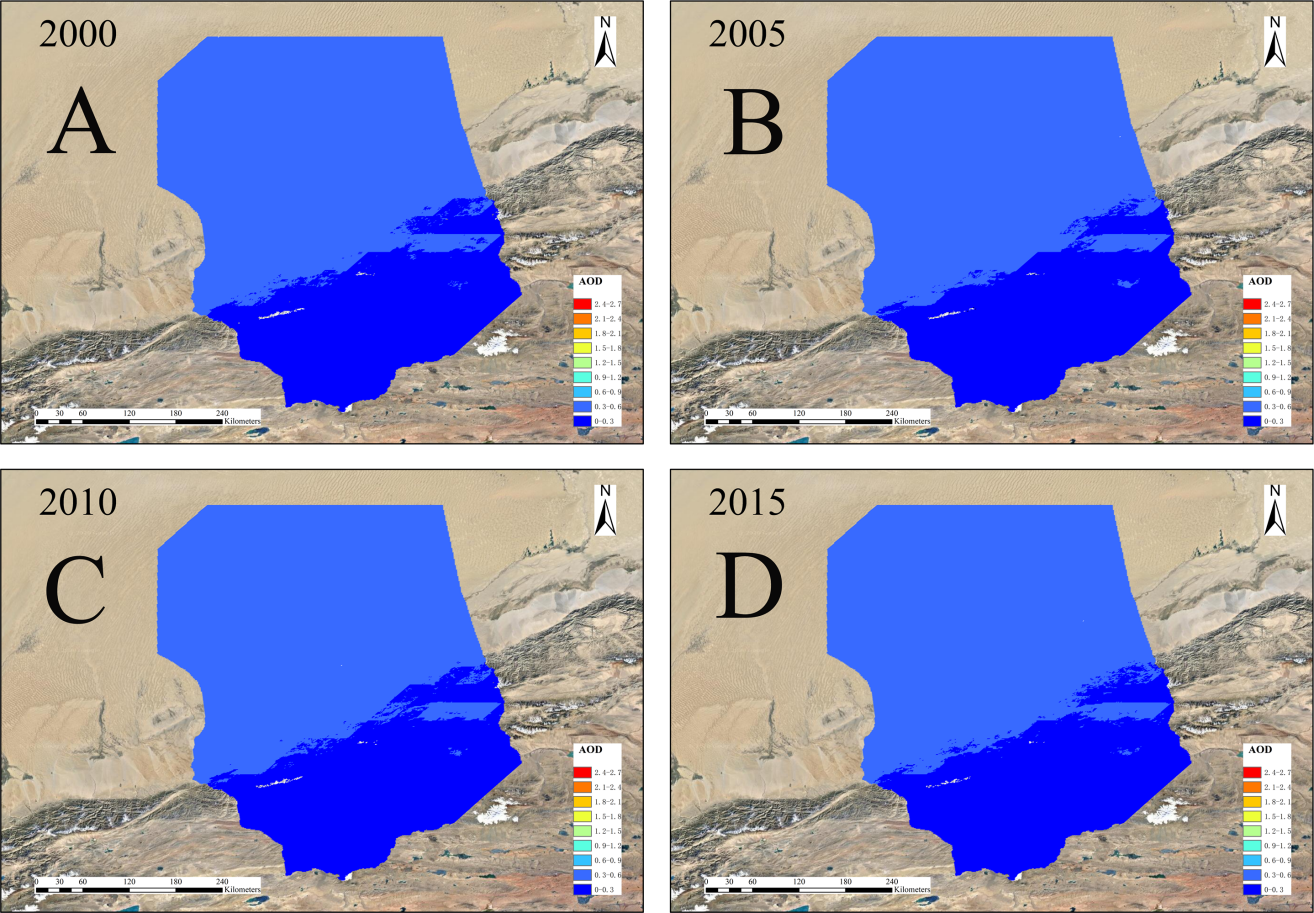
Annual average AOD in the Taklimakan Desert 2000/2005/2010/2015. (A–D) 2000, 2005, 2010, and 2015.

Figure 10 shows annual distribution of the average AOD from 2000 to 2015. The low-value areas from 2000 to 2015 always remained in the bottom of the Desert and its margins. Differences were observed in the range of high-value areas. The high-value areas in 2000 diffused from the central areas, and the high-value areas above the middle areas occupied half of the area. The range of high-value areas in 2005 was small and scattered, and the median range was concentrated. In 2010 and 2015, the high-value range significantly expanded. The maximum of AOD gradually rose from 2000, 2005, 2010 and 2015.

**Figure 11:**
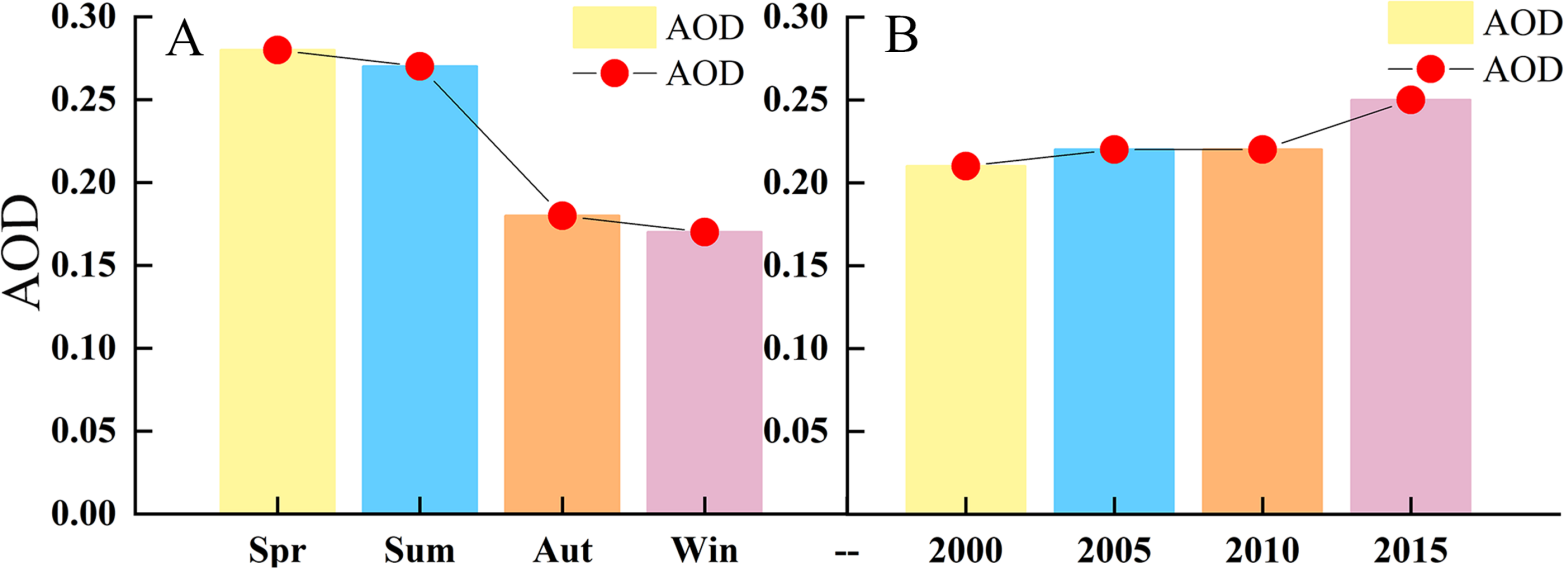
(A) Average seasonal AOD (B) Average annual AOD 2000/2005/2010/2015. The labels show AOD value, (A) seasons and (B) year.

As shown in Fig. 11A, the average AOD concentrations are highest in spring. As early summer is also the main season for frequent winds, the amounts of dust gradually decrease, while the fall and winter seasons are significantly lower. The average seasonal AOD decline rates were 3.6%, 33.3%, and 5.6% from spring to summer, from summer to autumn, from autumn to winter. The average AOD value of the four quarters has respectively decreased and from spring to winter the decrease in the AOD value is most obvious, as high as 39.3%. The downward trend increases from summer to autumn. This is reflected from the perspective that atmospheric conditions are more severe in spring than in autumn and winter and the air quality is relatively poor. The Taklimakan Desert has experienced significant seasonal differences in mean AOD over the years owing to decreases in certain meteorological factors (such as humidity), the average AOD value shows a significant downward trend from spring to winter.

As shown in Fig. 11B, the average AOD are relatively stable with a gradual increase to 2010 a large increase to 2015 (the AOD value is the annual averages with 5-year intervals). The AOD growth rates were 4.8%, 1.8%, and 16.7% from 2000 to 2005, from 2005 to 2010, from 2010 to 2015. Among them, the AOD values were highest in 2015 at 0.26. Overall, the annual increase rate was as high as 23.3%. When temperature increases, the local wind will produce thermal differences, due to uneven heating and temperature difference, leading to increased turbulence, Wind speed increases above a certain threshold, will project more sand and dust into the air, increasing of aerosol concentration. When the wind speed reduces, dust particles under the action of gravity settle back to land. Moreover, precipitation is extremely scarce. The groundwater content is very low and dust cannot be consolidated, so the accumulation of dust will cause the aerosol content to show an increasing trend. Therefore, wind speed is the key factor affecting the strength of dust weather and the change of dust aerosol concentration.

### The importance of environmental factors

**Figure 12:**
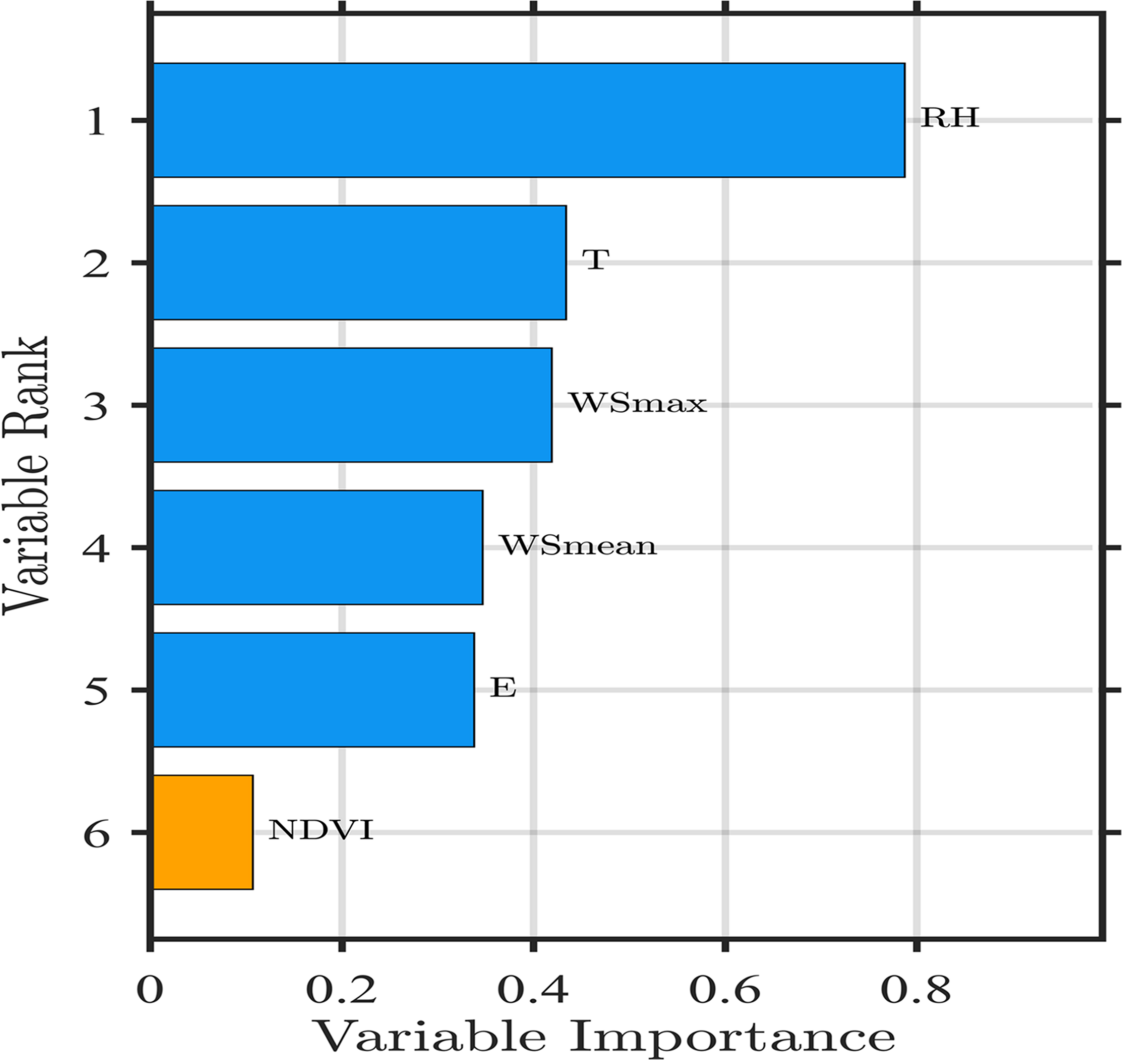
Random forest (RF) model simulation AOD renderings.

The predicted values from application of the RF model are shown in Fig. 12. They are highly consistent with the daily composite values of the MODIS AOD products with R2 values as high as 0.91. The impact of environmental covariates on AOD of the desert and its margins is ranked as follows: relative humidity > temperature > wind speed (max) > wind speed(mean)> evaporation> Normalized Difference Vegetation Index. RH has the greatest effect on AOD, followed by temperature, while NDVI has the smallest effect with weights of 0.798, 0.436, and 0.138, respectively. NDVI is not a major influencing factor for AOD, probably because the Taklimakan Desert is far from the ocean, cold and humid air currents and the Indian Ocean warm and humid air currents have difficulty reaching the desert, precipitation is extremely low, the sand blown by the wind is high, and vegetation has difficulty surviving. Conclusively, the dominant factor directly or indirectly affecting AOD in the Taklimakan Desert is RH. Temperatures, wind speeds, evaporation, and other factors work together to affect AOD. This study shows that the use of the RF model to estimate AOD from selected environmental covariates has good potential for application.

### Relationship between temperature and humidity in sandy weather in the desert and its margins

**Figure 13:**
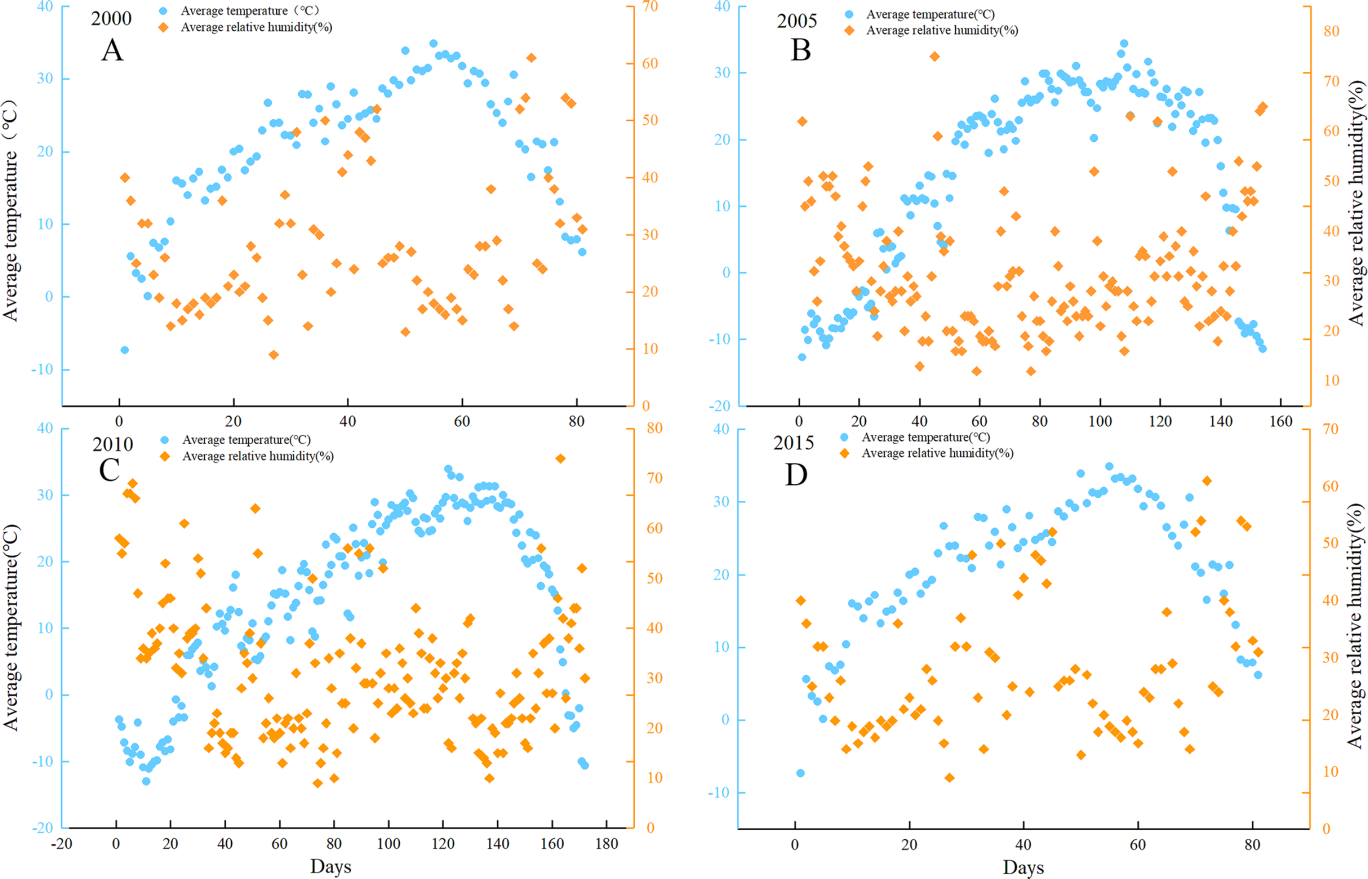
Distributions of the relationship between average temperature and average relative humidity in dust weather (2000 (A), 2005 (B), 2010 (C) and 2015 (D)). The blue line represents the average temperature, the orange line represents the average relative humidity, and the abscissa represents the number of days.

Based on the data of average temperature and average RH on days with dust from 2000 to 2015 provided by atmospheric environment observations and the Tazhong Desert Atmospheric Environment Observation Experimental Station of the China Meteorological Administration, we mainly analyzed temperature and humidity and showed that AOD is directly or indirectly affected by RH. In Fig. 13 (Note: the *X* axis represents the number of days where sand and dust weather occurred in different years). Dust weather refers to when strong wind at ground level picks up a large amount of dust, air turbidity increases, horizontal visibility is significantly reduced, is a common disastrous weather phenomenon. According to the definition of sand dust by China Meteorological Administration, it can be divided into floating dust, sand blowing and sand storm. The horizontal visibility of floating dust is less than 10 km, the horizontal visibility of sand blowing is less than or equal to 1 km to less than 10 km, and the horizontal visibility of sand storm is less than 1 km. The distance of horizontal visibility is used to determine the occurrence of dust weather shows that when dust weather occurred from 2000 to 2015 (this article mainly analyzes the frequency of dust weather), the average temperature had a strong negative relationship with average RH. We speculate that when dust becomes mobile, there is rapid drying at the surface and RH decreases very quickly. because moisture binds the dust grains and wind allows heat access between those dust and sand grains and everything dries out faster. Whereas, our result show that humidity and AOD are negatively correlated, thus AOD will increase result from dust weather. Here, we can consider the dust weather as an essential part of the increase in dust aerosols.

In addition, we use the simple Aridity Index created by De Martonne (calculated by two climate factors, temperature and precipitation) to indirectly testify whether or not a change in RH has occurred, thus affecting AOD. By using the annual temperature and precipitation data of 2000, 2005, 2010 and 2015 (excluding the missing data), we obtained the Aridity index of 10.01, 10.01, 10.00 and 10.00 respectively, showing an overall decreasing trend, indicating that evaporation was more intense and precipitation was very rare, so RH would also decrease. According to the meteorological data of the Taklimakan Desert, there has been a upward trend of dusty weather in recent years. Moreover, wind speed is a key factor affecting the intensity of sand and dust weather. Thus, we can confidently assume that if in the future, the occurrence of dusty weather (dust, sand, etc.) increases, AOD may show an upward trend; however, the increase or decrease in AOD is still affected by various conditions.

### Correlation analysis between AOD and meteorological variables or factors

**Figure 14:**
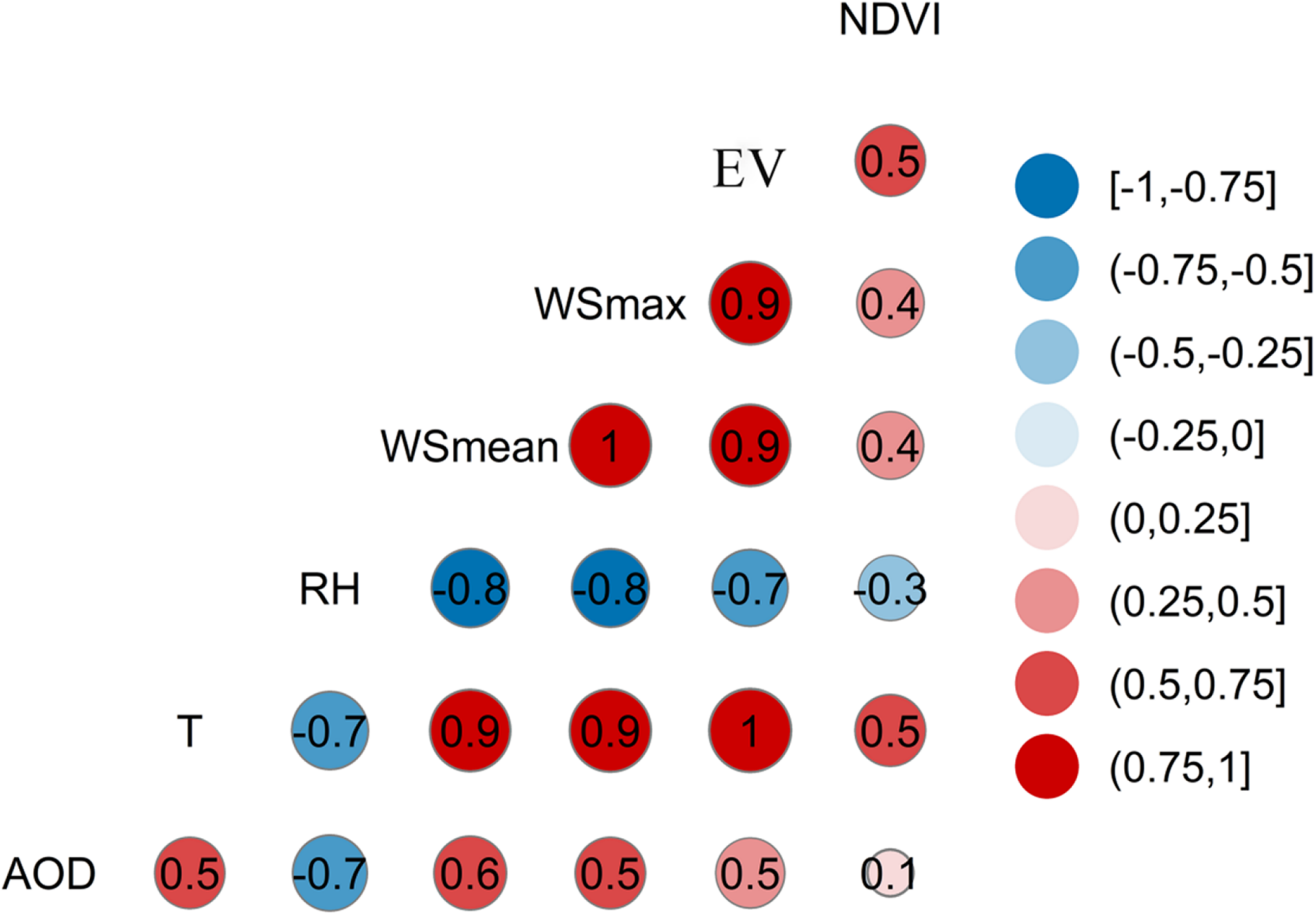
Correlation coefficients between AOD and meteorological variables in the Taklimakan Desert.

Figure 14 presents the correlations between AOD and various meteorological elements (factors). NDVI exhibits the smallest correlations with other variables. Because there is little accumulation of water in the desert and its margins, the desert is highly mobile, preventing the establishment and survival of vegetation. Vegetation growth is severely challenged, and vegetation coverage hardly expands. Monthly NDVI in 2000, 2005, 2010, and 2015 showed little seasonal variation. Therefore, NDVI is not a major factor affecting dust aerosols. In terms of meteorological elements, there is a clear negative correlation between relative humidity (RH) and AOD; their correlation coefficient is the greatest at 0.7, followed by mean wind speed (WSmean) at 0.6. The correlation coefficients of atmospheric temperature, maximum wind speed, evaporation, and AOD are all 0.5. Overall, AOD are most affected by Meteorological factors (Relative humidity).

## Discussion

The spatial resolution of the MCD19A2-MODIS AOD data reaches 1 km, and it compensates for the shortcomings of the dark target method and dark blue algorithm associated earlier observations. With regard to dark surfaces, the dark target method has good accuracy; for bright surfaces, its accuracy is better than that of the dark blue algorithm. However, regardless of the aerosol product, verification by actual measurement is essential. This is because of the underestimation of RS products, which is a common problem because RS data only simulate or retrieve the real surface and the value cannot be consistent with the real value. This is also the main source of error. Hence, we must verify the RS products. Earlier, MOD08 products were used to study the Taklimakan Desert (Kim et al., 2007); however, underestimation, compared with the measured values, was observed. The products used in this study are more accurate.

Real-time ground monitoring cannot fully reflect the change trends and spatial distribution characteristics of atmospheric environmental quality in a certain area and range. Under traditional conditions, the analysis and research of atmospheric aerosols mostly comprise real-time monitoring on the ground. However, this approach can only reflect AOD changes and distribution characteristics over a small distance near the monitoring point. It is difficult to achieve real-time continuity in space, and the cost of data acquisition is high. Therefore, it is difficult to meet research needs. However, aerosol RS technology can compensate and solve the problem of a lack of measured data and uneven spatial distribution. The RS technology has the characteristics of high efficiency, large scale, and low cost. It can reflect the distribution characteristics, diffusion dynamics, and propagation paths of air pollutants at large scales such that we can obtain AOD data on a large regional scale and at high time resolution (Della Ceca et al., 2018; Gupta et al., 2016). In this research, the NDVI used MODIS series data and authors used MODIS AOD data. NDVI is a 250-m synthetic product dataset that is produced every 16 days. AOD is a daily synthetic product dataset, and AOD product datasets are synthesized by averages, which can avoid external environmental effects well and thus facilitate more realistic data.

Research and analysis of the cyclical characteristics of MODIS AOD products show that the average AOD maintained a steady and slow rise from 2000, 2005 and 2010 and showed a large increase in 2015. Combined with the analysis of surface meteorological data, this may be related to the increase in the number of high winds and dusty events. Based on the analysis of seasonal changes in the average AOD in the four periods, the AOD in spring is the highest, probably owing to the development of updrafts when local surfaces become warm. The sand particles on the desert and dust on bare surfaces cause the concentration of particulate matter in the atmosphere to increase at a large scale, leading to maximum AOD values in the study area in spring. Then, the AOD concentration value gradually decreases and reaches a minimum in winter (Che et al., 2012).

The RF model was used to simulate the effect of AOD values using six environmental covariates: RH, T, WSmax, WSmean, EV, and NDVI. Because the Taklimakan Desert is remote with no human interference, dust aerosols are mainly constrained by natural conditions. The results showed that RH contributed most to the change in AOD, followed by temperature. NDVI contributed the least, probably because of the extremely scarce vegetation in the Taklimakan Desert and lack of plant cover in nearly the entire area. Moreover, the highly mobile sand layer hinders the spread of already sparse plants. This lack of diffusion indicated that NDVI cannot be the factor affecting AOD the most. However, the boundaries in Figs. 9 and 10 between low and high AOD are so stable, it might be related to low vegetation cover. The NDVI is not a fine product to express the vegetation coverage, it only reflects one of the important parameters of crop growth and nutritional information. In the lower vegetation cover region, the spatial distributions in AOD on the ground might be highly relatable to the presence of vegetation. The Taklimakan Desert has a typical warm temperate with continental arid climate. The temperature is high throughout the year, leading to strong evaporation and high dryness of the sand surface. Thus, temperature is an important factor affecting AOD.

Dusty weather in the Taklimakan Desert has increased over the past 20 years. Sand, dust, and even tornadoes can contribute to AOD changes. Moreover, long-distance transport of dust aerosols in East Asia must be considered (Gu et al., 2016; Guo & Yin, 2015). There are many other factors affecting sandstorms, such as polar vortexes, evaporation speed, and precipitation. In addition to weather conditions, atmospheric stability and wind field characteristics may cause sandstorms, which require our attention.

Due to the limitations of solar photometer observations, mainly due to spatiotemporal limitations, the number of measured samples that can be matched with RS satellites is not very large. The correlation analysis between MODIS AOD data and ground-based measured data can only provide partial reference help. It should be from point to the surface, and more measured data are needed for verification.

Aerosol optical depth is affected not only by atmospheric pollutants and daily meteorological factors but also by cumulative effects of various environmental variables over the preceding few days. Further, this study has certain limitations. Some downloaded MODIS AOD data are missing, and some information may be lost. Through analysis, it was found that RH is the most important factor affecting the change in AOD. in addition, On a large scale, the importance of RH still needs to be further studied.

## Conclusion

The average monthly AOD value in the Taklimakan Desert showed a significant trend of increasing and then decreasing with a “single peak” curve. Moreover, the average AOD value in the four seasons showed obvious seasonal change characteristics; from 2000 to 2015, the AOD in spring occupied first place (0.28), the value in summer came second (0.27), the value in winter was minimum (0.17). In spring, the air pollution in the Taklimakan Desert was most serious. After summer, the AOD value significantly decreased and remained relatively stable. The annual average AOD value is 0.23, and the interannual change exhibited a gradual increasing trend. From 2000 to 2005, it increased slowly that the AOD value from 0.21 to 0.22; from 2005 to 2010, it was relatively stable (from 0.220 to 0.224); from 2010 to 2015, the AOD value clearly and rapidly increased that the value from 0.22 to 0.26. These result indicated that air pollution in the Taklimakan Desert and its margins has intensified in the last 20 years, particularly in the last 5 years. The deterioration of the air environment is more prominent and severe. From the correlation analysis, it was found that the correlations between AOD and various meteorological elements were different. Among them, AOD and RH showed a significant negative correlation, which was largest at 0.7. The effect of RF model on AOD showed better correlation, *R*^2^ = 0.91; RH had the most significant effect on AOD.

## Supplemental Information

10.7717/peerj.10542/supp-1 Supplemental Information 1 Raw data.

## Additional Information and Declarations

### Data Availability

The following information was supplied regarding data availability: Meteorological and AOD data are available as a Supplemental File.
